# MTMR4 Is Required for the Stability of the *Salmonella*-Containing Vacuole

**DOI:** 10.3389/fcimb.2016.00091

**Published:** 2016-08-30

**Authors:** Wei X. Teo, Markus C. Kerr, Rohan D. Teasdale

**Affiliations:** Institute for Molecular Bioscience, University of QueenslandBrisbane, QLD, Australia

**Keywords:** *Salmonella*, Phosphotidylinositols, myotubularin, autophagy, *Salmonella*-containing vacuole

## Abstract

The intracellular pathogen *Salmonella enterica* servovar Typhimurium (*S.typhimurium*) modulates the host cell's phosphoinositide (PI) metabolism to establish its intracellular replicative niche, the *Salmonella*-containing vacuole (SCV). Upon invasion, phosphoinositide 3-phosphate (PI(3)P) and other early endosomal markers are rapidly recruited to and remain associated with the SCV throughout its early maturation. While the phosphoinositide 3-phosphatase myotubularin 4 (MTMR4) has an established role in regulating autophagy and cellular PI(3)P-content, two processes associated with the intracellular survival of *S. typhimurium*, a direct role for MTMR4 in *Salmonella* biology has not been examined. Here we demonstrate that GFP-tagged MTMR4 is recruited to the SCV and infection of cells depleted of endogenous MTMR4 results in a decrease in viable intracellular *Salmonella*. This reflects a significant increase in the proportion of SCVs with compromised integrity, which targets the compartment for autophagy and consequent bacterial cell death. These findings highlight the importance of PI(3)P regulation to the integrity of the SCV and reveal a novel role for the myotubularins in bacterial pathogenesis.

## Introduction

*Salmonella* is a Gram-negative facultative intracellular pathogen and a major cause of disease in humans (Haraga et al., 2008). The two predominant human pathogenic strains are *Salmonella enterica* serovar typhi, responsible for typhoid fever, and *S. enterica* servoar typhimurium, a causative agent of human gastroenteritis (Haraga et al., 2008). Once ingested, *Salmonellae* traverse the digestive tract and invade non-phagocytic epithelial cells lining the intestinal walls. Bacterial effector proteins are translocated directly into the host cells by one of two Type 3 Secretion Systems (T3SSs), to manipulate host membrane trafficking and cytoskeletal elements, initiating macropinocytosis and uptake of the pathogen into the cell (Kubori et al., 1998; Zhou and Galan, 2001). Whilst at least 40 effector proteins are translocated by the *Salmonella* Pathogenicity Island 1 (SPI1)-T3SS, prominent amongst these is SopB, a phosphatidylinositol phosphatase with sequence similarity to both mammalian phosphatidylinositol 4-phosphatase and phosphatidylinositol 5-phosphatase (Norris et al., 1998).

Phosphotidylinositols are an important class of lipid signaling molecules that can be singly or multiply phosphorylated on their inositol group to yield 7 spatio-temporally regulated phoshoinositides that are integral to a variety of cellular processes (Balla, 2013). Of these, phosphatidylinositol 3-phosphate (PI(3)P) and phosphatidylinositol 3,5-bisphosphate (PI(3,5)P_2_) are primarily responsible for the regulation of traffic within the endosomal pathways (Di Paolo and De Camilli, 2006; Kerr et al., 2010). Following invasion, *Salmonella* alters the encompassing macropinosome to generate a replicative niche known as the *Salmonella*-containing vacuole (SCV). Concomittantly, the *Salmonella* Pathogenicity Island 2 T3SS (SPI2-T3SS) is activated, facilitating pathogen survival and replication (Figueira and Holden, 2012). Early in development, the SCV associates with organelles of the endosomal system, acquiring markers such as EEA1, SNX1 (Bujny et al., 2008), PI(3)P, and Rab5 (Dai et al., 2007; Bakowski et al., 2010). PI(3)P in particular is crucial to the stability and integrity of the SCV as intracellular *Salmonella* treated with PI(3)-kinase inhibitor, wortmannin, escape from the SCV and replicate within the cytoplasm unchallenged (Brumell et al., 2002; Scott et al., 2002). In the later stages of infection, SCV maturation is characterized by the formation of tubular protrusions called *Salmonella* Induced Filaments (SIFs), as well as the loss of PI(3)P and the acquisition of late endosomal markers such as LAMP1 and Rab7 (Knodler and Steele-Mortimer, 2005). Perturbing this maturation through inhibition of the phosphoinositide 5-kinase, PIKfyve, blocks SIF formation and has significant impact on the intracellular growth of the pathogen (Kerr et al., 2010). These observations highlight the tight spatiotemporal coordination of PI(3)P and PI(3,5)P_2_ on the SCV and demonstrate that alteration of either will ultimately influence the intracellular fate of the pathogen.

The 3′-dephosphorylation of PI(3)P and PI(3,5)P_2_ to phosphatidylinositol (PI) and phosphatidylinositol 5-phosphate (PI(5)P) is governed by the myotubularin (MTMR) family (Robinson and Dixon, 2006). The 14 myotubularins are characterized by the presence of a signature phosphatase domain, for which 6 are inactive due to mutations within the catalytic site. Of the 8 with an active phosphatase domain, MTMR3 and MTMR4 are further distinguished by a C-terminal FYVE domain (Lorenzo et al., 2006) but only MTMR4 is localized to PI(3)P-containing early endosomes, with MTMR3 localized to the cytosol (Lorenzo et al., 2006; Naughtin et al., 2010). MTMR4 can dephosphorylate PI(3)P *in vitro* and is recruited to the both early and recycling endosomes where it has been shown to influence the PI(3)P levels on these organelles (Zhao et al., 2001; Lorenzo et al., 2006; Naughtin et al., 2010). The capacity for MTMR4 to dephosphorylate PI(3,5)P_2_ remains unclear with the only evidence that MTMR4 immunoprecipitates appear to dephosphorylate PI(3,5)P_2_ (Naughtin et al., 2010).

Here we demonstrate that RNAi-mediated depletion of MTMR4 perturbs the intracellular growth of *S. typhimurium*, further highlighting the pathogen's dependence upon the 3-PIs. Specifically, we demonstrate that MTMR4 is recruited to PI(3)P-rich SCVs and report dysregulation of PI(3)P dependent stages of SCV maturation in MTMR4-depleted cells. This destabilizes the SCV leading to release of the pathogen into the cytoplasm. Concommitantly, we observe an elevation in the autophagy pathway and ultimately the destruction of the cytoplasmic *Salmonella* by the host cell innate immune system.

## Materials and methods

### Constructs and reagents

HA-MTMR4, GFP-MTMR4, GFP-MTMR3, GFP-LC3, myc-2^*^ML1N were as described previously in Walker et al. (2001), Birmingham et al. (2006), Lorenzo et al. (2006), Naughtin et al. (2010) and Li et al. (2013). mCherry-2^*^ML1N was obtained by performing restriction digest using restriction enzymes BglII and EcoRI on myc-2^*^ML1N to obtain the open reading frame of 2^*^ML1N and subcloned into mCherry-C1 following standard protocols. Monoclonal antibodies against EEA1 (610457, 1:100) and SNX1 (611483,1:100) were supplied by BD Bioscience. Monoclonal antibodies against the haemagglutinin epitope (MMS-101P, 1:1000) (YPYDVPDYA), myc epiptope (9B11, 1:2000), galectin-8 (ab109519,1:100) and p62 (R&D, MAB8028, 1:100) were supplied by Cell Signaling Technology, Abcam and R&D systems respectively. Monocloncal antibodies against β-tubulin (T3526, 1:2000) was purchased from Sigma Aldrich. Rabbit polyclonal antibodies against LC3 (NB100-2220) and GFP (A-6455, 1:500) were purchased from Novus biological and Molecular Probes (Invitrogen). Secondary antibodies and dextran-fluorescent conjugates were purchased from Life Technologies. *S. Typhimurium* strains used include the wild-type strain SL1344 (Hoiseth and Stocker, 1981) and the isogenic derivatives RFP-SL1344 (Birmingham et al., 2006) and SopB-deficient SL1344 (Mallo et al., 2008). Rapamycin, Wortmannin and 3-MA were purchased from Sigma Aldrich.

### Cell culture and transfection

A431 cells and Hela cells stably expressing GFP-LAMP1 (Kehl and Hensel, 2015) were maintained in Dulbecco's modified Eagle's medium (DMEM) supplemented with 10% (v/v) fetal bovine serium with 2 mM l-glutamine (Invitrogen) in humidified air/atmosphere (5% CO_2_) at 37°C. Cells were transfected using Lipofectamine 2000 as per manufacturer's instructions (Life Technologies)

### shRNA-mediated knockdown

pGIPZ small hairpin RNA (shRNA) plasmids (Thermo Scientific) used included non-silencing control shRNA: RHS4346; human MTMR4 shRNAs (shRNA #1: V2LHS_375815), were supplied by the Institute for Molecular Bioscience Life Science Automation (LISA) Facility. A431 cells were transduced with shRNA particles in the presence of 8 μg/ml polybrene (Milipore) for 4 h in serum free media before incubating them overnight in normal growth media. Transduced cells were selected for with DMEM containing 1 μg/ml puromycin (Sigma Aldrich) for 1 week.

### Quantitative RT-PCR

RNA from cells was extracted according to the manufacturer's directions (Sigma Aldrich). One microgram total RNA was used to produce cDNA using oligdT primers and Superscript III (Invitrogen). Quantitative RT-PCR was conducted on samples following shRNA treatment and analyzed in triplicate. The housekeeping gene GAPDH was used as an internal control to calculate the ΔCT for each sample. Primers for MTMR4 were targeted. MTMR4 expression was quantified using TaqMan gene expression assays as per manufacturer instructions (Applied Biosystems) in 96 well plate format.

### Dextran uptake

Endosomes were labeled with fluorescent dextran by culturing live cells in the presence of 100 μg/ml dextran conjugated to tetramethylrhodamine for 15 min before being washed thoroughly with excess media and fixed for further analysis.

### *Salmonella* infection and gentamicin protection assay

*Salmonella* infections were performed as previously described (Yang et al., 2015). Briefly, *S. typhimurium* (SL1344) was cultured in Luria Broth (LB) overnight at 37°C with shaking (225 rpm) followed by dilution into 3 ml of fresh LB (1:31) and continued culture for a further 3.5 h. 1 ml of the resultant culture was then centrifuged at 13,000 rpm for 1 min and resuspended in 10 ml of phosphate-buffered saline (PBS) and the optical density (OD) measured. The appropriate bacterial dilution was prepared in normal growth media and HeLa cells infected with a multiplicity of infection (MOI) of 1.0 unless otherwise stated. After 30 min of infection, the cells were washed three times and incubated in normal media containing 100 μg/ml gentamicin. At 1.5 h p.i., the gentamicin concentration was reduced to 10 μg/ml for the remaining duration of the experiment.

### Colony forming unit assay

Colony forming unit assay was performed as previously described (Kerr et al., 2010). Briefly, infected cells were washed 3 times with PBS and lysed in 1X PBS containing 0.25% SDS. The resultant lysate were serially diluted and plated onto LB agar plates containing 100 μg/ml Ampicillin. The plates were incubated at 37°C for 24 h and resultant colonies counted to determine the number of Colony Forming Units (CFU).

### Time-lapse videomicroscopy

For live imaging, cells were seeded on 35 mm glass-bottom MatTek dishes and subsequently imaged on a Zeiss LSM 710 confocal microscope using a 63 X or 100 X oil immersion objective and maintained at 37°C and imaged over the time-course in a 5% CO2 humidified chamber.

### Immunofluorescence and confocal microscopy

Cells fixed with 4% paraformaldehyde were permeabilized with 0.1% triton X-100 for 10 min, and blocked in 2% BSA (in 1xPBS). Primary antibodies were incubated for 1 h at room temperature. After incubation, cells were washed three times with blocking reagent and incubated with Alexa-Fluor-488-conjucated, Alexa-Fluor-547-conjugated and Alex-Fluor-647-conjugated secondary antibodies (1:400) Cells were also labeled with DAPI, washed three times with 1xPBS and mounted onto glass slides using mounting Media. Single-plane or stacked images were obtained using a Zeiss LSM 710 upright Microscope and a 40 X oil or 63 X oil immersion objective.

### Western immunoblotting

Cell lysate samples were subjected to bicinchoninic acid (BCA) assay (Thermo Scientific) to determine total protein concentration. Twenty microgram of protein per sample were resolved by SDS-PAGE transferred onto Immobilon-P membrane (Millipore) according to the manufacturer's instructions. Western immunoblotting using ECL was performed as per manufacturer's instructions.

### Quantification of *Salmonella* growth

Intracellular *Salmonella* growth was analyzed and quantified by FIJI. Briefly, the “subtract background” functionality was applied and images thresholded to an intensity of 155 (8-bit image) and quantified through the “Analyze Particles” feature. The resultant particle count is represented in arbitrary units. All results were tabulated and presented using GraphPad software version 6.

### Quantification of markers acquisition by SCVs

Maximum projections images were captured using a Zeiss LSM710 confocal laser scanning microscope under 63 × magnification and the number of endosomal markers positive SCVs were quantified using FIJI. Endosomal markers overlapping SCVs were scored as positive. All results were tabulated in GraphPad Prism software version 6 and represented as a percentage out of 100.

### Statistical analysis

Data were analyzed using Student's *T*-test, performing pairwise analysis to control where appropriate. A *p*-value of less than 0.05 was considered statistically significant. All analyses were performed using GraphPad Prism software version 6.

## Results

### PI(3,5)P_2_ is present on the mature *Salmonella*-containing vacuole

**Figure 1:**
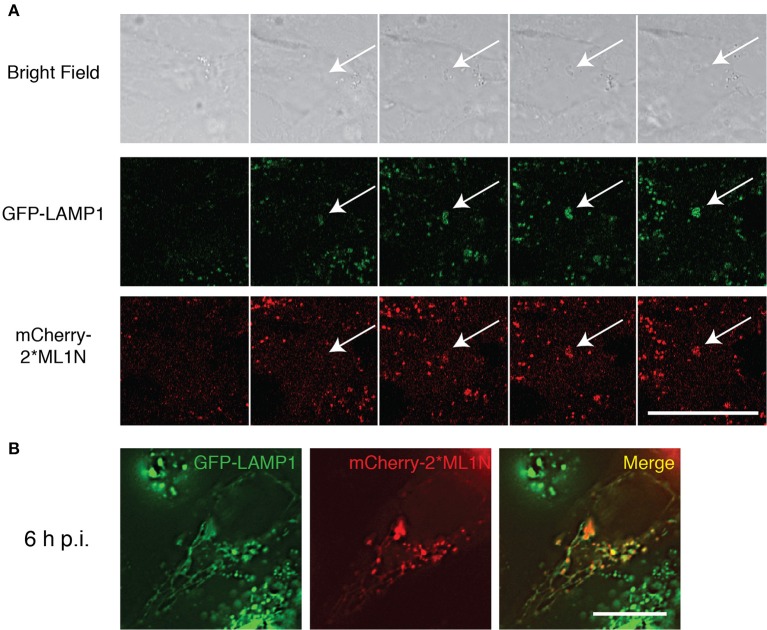
**Recruitment of PI(3,5)P_2_ to the SCV and Sifs. (A)** Time-lapse video microscopy of WT-SL1344 infected HeLa cells stably expressing GFP-LAMP1 and transiently expressing mCherry 2^*^ML1N. Montage representative of a 90 min movie captured using Zeiss LSM710 FCS scanning confocal microscope. **(B)** HeLa cells stably expressing GFP-LAMP1 cells and transiently expressing mCherry-2^*^ML1N were infected with WT-SL1344 and imaged 6 h p.i live on Zeiss LSM710 FCS scanning confocal microscope. Scale bar: 20 μm. White arrows indicate SCV.

The transition of early to late endosomal markers as the SCV matures implies the conversion of PI(3)P to PI(3,5)P_2_. We demonstrated that inhibition of PIKfyve disrupts the formation of SIFs and the intracellular replication of *S. typhimurium* (Kerr et al., 2010), however, due to the absence of a PI(3,5)P_2_-specific probe at the time, we were unable to directly monitor the presence of PI(3,5)P_2_ accumulation on the SCV. Recently a PI(3,5)P_2_-specific probe was reported (Li et al., 2013) and we therefore sought to monitor the association of PI(3,5)P_2_ with the SCV. Hela cells stably expressing GFP-LAMP1 were transfected with mCherry-2^*^ML1N and infected with late log-phase *S. typhimurium* (WT-SL1344). Time-lapse videomicroscopy revealed a concomitant accumulation of mCherry-2^*^ML1N and LAMP1 on the SCV from 60 min post-infection (p.i.) (Figure 1A). The final stages of SCV maturation include the formation of extensive LAMP1-positive tubular structures known as SIFs, with which mCherry-2^*^ML1N remains associated at 6 h p.i (Figure 1B), validating the model that the SCV transitions from a PI(3)P-positive compartment to become PI(3,5)P_2_-positive as it matures. Given that we demonstrated that the SCV is only transiently enriched with PI(3)P (Kerr et al., 2010), we speculated that concurrent with its conversion to PI(3,5)P_2_, SCV-PI(3)P may also be dephosphorylated to PI by the endosome-associated myotubularin phosphatase MTMR4 to ensure complete maturation of the SCV.

### MTMR4 regulates PI(3)P content on early endosomes but does not impact PI(3,5)P_2_

**Figure 2:**
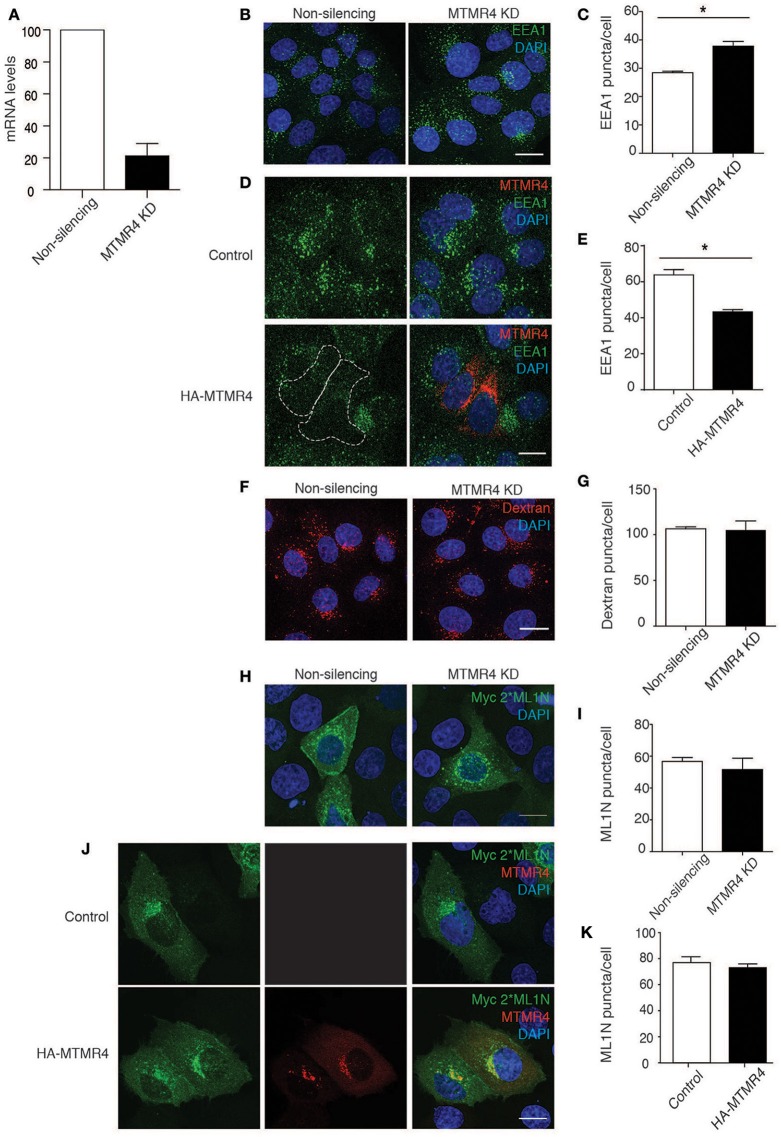
**MTMR4 regulates PI(3)P endosomal levels but not PI(3,5)P_2_. (A)** A431 cells were transduced with shRNA targeting MTMR4 and the knockdown efficiently confirmed using RT-PCR. **(B)** MTMR4-depleted cells were fixed and immunolabelled with EEA1, counterstained with DAPI. **(C)** The number of EEA1 puncta was analyzed. **(D)** A431 cells were seeded and transfected with HA-MTMR4 or HA-empty vector, fixed, and immunofluorescence performed using antibodies against EEA1. **(E)** Quantification of EEA1 puncta following overexpression of HA-MTMR4. **(F)** MTMR4-depleted cells were pulsed with dextran conjugated to tetramethylrhodamine for 30 min before thorough washing with excess media and fixed, counterstained with DAPI. **(G)** Quantification of dextran puncta. **(H)** MTMR4 depleted cells were transfected with Myc-2^*^ML1N construct, immunolabelled and quantified in **(I)**. **(J)** A431 cells were co-transfected with MTMR4 or empty vector and Myc-2^*^ML1N, fixed, immunolabeled and puncta quantified in **(K)**. Data representative of three independent experiments (*N* = 3; at least 25 cells counted per condition; Error bar denote ± S.E.M., ^*^P < 0.05, Scale bar: 20 μm).

To examine how MTMR4 regulates intracellular PI(3)P and/or PI(3,5)P_2_ concentrations, shRNA-MTMR4 depleted A431 cells were generated and validated using RT-PCR (Figure 2A). MTMR4 knockdown cells, as well as those overexpressing HA-MTMR4, were fixed and immunolabelled for endogenous PI(3)P-binding EEA1 (Simonsen et al., 1998) or the PI(3,5)P_2_-binding probe Myc-2^*^ML1N (Li et al., 2013). Cells depleted for MTMR4 presented a 25% increase in the total number of EEA1-positive puncta relative to non-silencing control cells (Figures 2B,C). In contrast, HA-MTMR4 overexpression decreased the total number of EEA1-positive puncta by 30% relatively to the mock-transfected cells (Figures 2D,E). To determine if the observed increase in EEA1 labeling in MTMR4-depleted cells reflected perturbation in overall number of endosomes, a 10,000 MW dextran-tetramethylrhodamine (TR) uptake assay was performed. A 30 min pulse-chase revealed no significant difference in the number of dextran-TR positive endosomes in the MTMR4-depleted cells when compared to the non-silencing control cells, indicating no significant impact on the number of endosomes formed within these cells (Figures 2F,G). These observations are consistant with the documented activity of MTMR4 (Lorenzo et al., 2006; Naughtin et al., 2010). Several members of the myotubularins family have been implicated to regulate PI(3,5)P_2_ (Robinson and Dixon, 2006) althought to date no direct evidence exists that PI(3,5)P_2_ is a substrate for MTMR4. To determine if MTMR4 modulates intracellular PI(3,5)P_2_, MTMR4 depleted or control cells were singly or co-transfected with either myc-2^*^ML1N and/or HA-MTMR4. No differences in fluorescent intensities and subcellular distributions of myc-2^*^ML1N in either cells depleted for MTMR4 or ectopically expressing MTMR4 were observed (Figures 2H–K). Taken together, these results indicate that MTMR4 negatively regulates PI(3)P content on early endosomes, but has no direct role in regulating PI(3,5)P_2_ distribution in the cell.

### MTMR4 is recruited and regulates early SCV maturation

**Figure 3:**
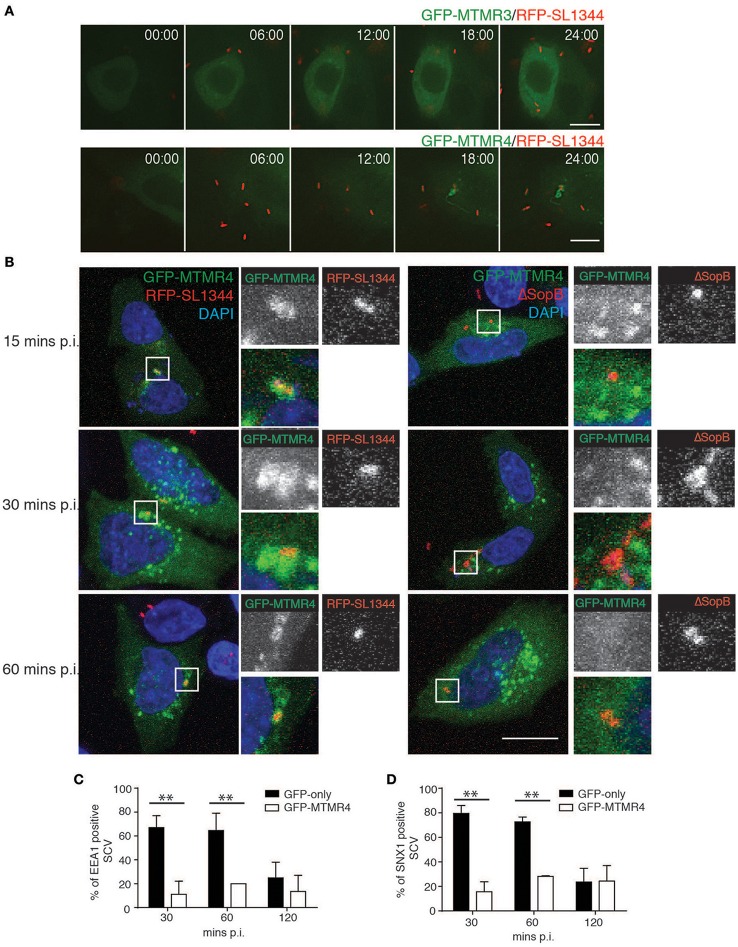
**MTMR4 is associated with the SCV. (A)** Time-lapse video microscopy of A431 cells transfected with GFP-MTMR4 or GFP-MTMR3 and infected with RFP-SL1344. Montage is representative of a 25 min movie captured using Nikon Deconvolution microscope. **(B)** A431 cells were transfected with GFP-MTMR4 and infected with either RFP-SL1344 or ΔSopB-SL1344. MTMR4-positive *Salmonella* were then probed for the presence of EEA1 or SNX1 and quantified over a time course assay **(C,D)** respectively. (*N* = 3; Error bar denote mean ± S.D., Scale bar: 20 μm, ^**^p < 0.005).

During *Salmonella* infection, PI(3)P has been observed to first accumulate on membrane ruffles and subsequently remains associated with the SCV throughout its early biogenesis (Scott et al., 2002). To determine if MTMR4 is associated with the nascent SCV, A431 cells were transfected with GFP-MTMR4 or GFP-MTMR3 for 18 h before being infected with a wildtype *S. typhimurium* strain (SL1344) expressing RFP (RFP-SL1344) at a MOI of 1. Time-lapse video microscopy demonstrated that GFP-MTMR4 but not GFP-MTMR3 associated with the nascent SCV as early as 15 min p.i. (Figure 3A). To dissect the mechanism of MTMR4 recruitment further, we infected GFP-MTMR4 expressing cells with a SopB-deletion mutant strain reported to be deficient in accumulation of PI(3)P on its SCV (Mallo et al., 2008). Indeed, MTMR4 was not recruited to the ΔSopB-SL1344 SCVs even at 1 h p.i (Figure 3B).

To examine the potential for MTMR4 in *Salmonella* invasion, an invasion assay was performed by infecting cells ectopically expressing GFP-MTMR4 with RFP-SL1344 at a MOI~5 for 10 min before fixing the cells. Anti-*Salmonella* LPS antibodies were used to probe for extracellular *Salmonella* in unpermeabalised cells, which revealed no difference the invasion rates between MTMR4 overexpressing (34.4% ± 1.99% internalized bacteria) or control cells (34.61 ± 1.66% internalized bacteria), confirming that MTMR4 does not play a role in bacterial invasion. Although, the early stages of an SCV appears to share properties in common with constitutive macropinosomes, including recruitment of early endosomal markers, SNXs, and PI(3)P, it is significantly modified by secreted *Salmonella* effectors so that the PI(3)P accumulates and remains elevated for up to 90 min p.i on individual SCVs (Hernandez et al., 2004). We next assessed the impact MTMR4 has on the early maturation of the SCV. GFP-MTMR4 expressing cells were infected with RFP-SL1344 for 30 min, washed and incubated with 100 μg/ml of gentamycin for 1 h to kill extracellular *Salmonella* before incubating the cells further in 10 μg/ml gentamycin. The samples were fixed, immunolabelled with antibodies specific for EEA1 or SNX1 followed by a fluorescently-conjugated secondary antibody and examined using confocal microscopy to quantify recruitment of these proteins to individual SCV (Figures 3C,D). At 30 and 60 min p.i, the majority of SCVs containing RFP-SL1344 in GFP-expressing control cells were associated with early maturation markers EEA1 and SNX1 which then decreased by 120 min as the SCV undergoes maturation. In contrast, ectopic expression of GFP-MTMR4 resulted in the MTMR4-positive SCVs remaining void of any early maturation markers throughout the 120 min time period examined (Figures 3C,D). Here we demonstrate that the elevated expression of MTMR4 decreases the normal recruitment of PI(3)P-dependent endosomal proteins during the early SCV maturation stages presumably due to the excess phosphatase efficiently converting PI(3)P to PI.

### Depletion of MTMR4 interferes with early survival of intracellular *Salmonella*

**Figure 4:**
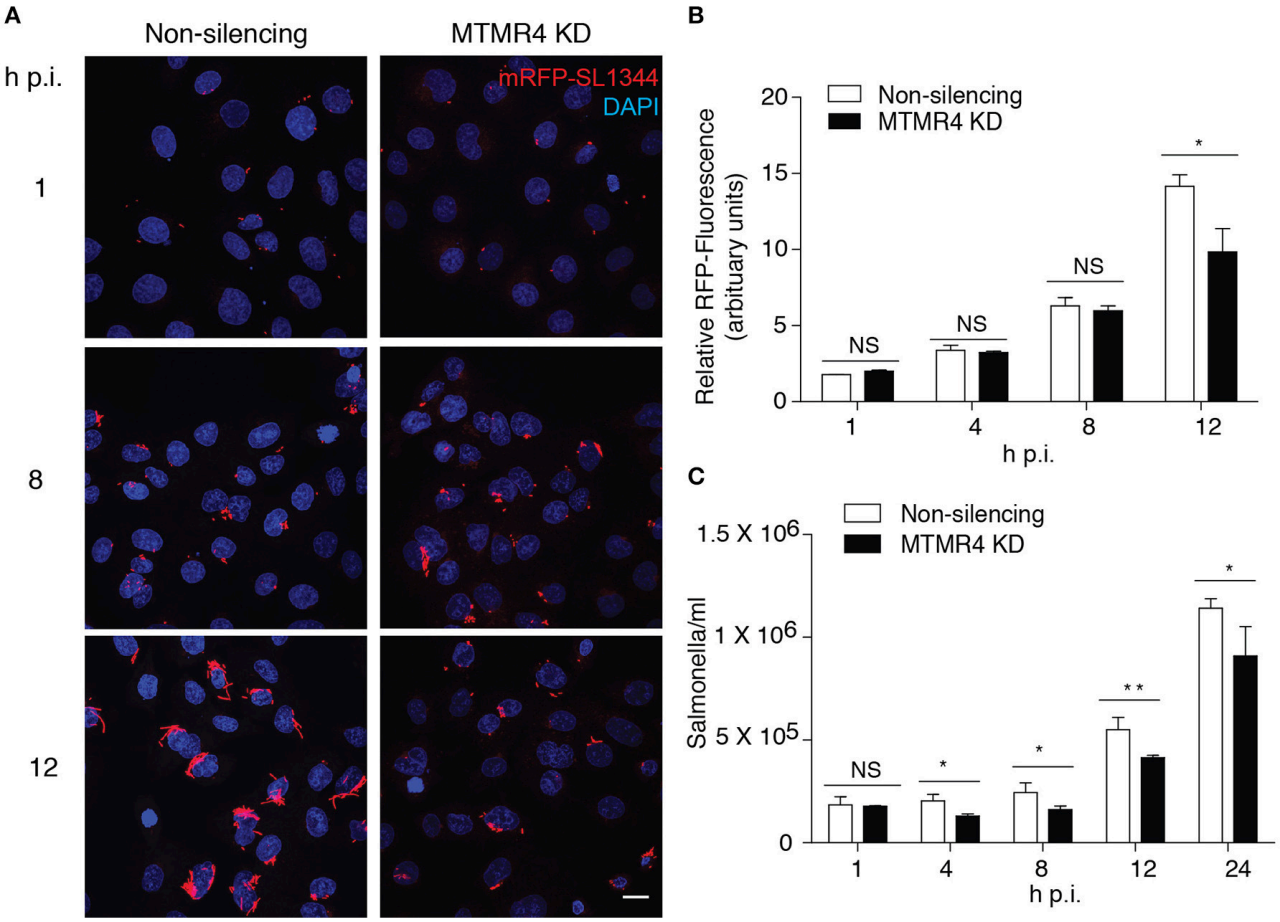
**MTMR4 depletion renders cells less permissible to SCV growth. (A)** MTMR4-depleted A431 and control cells were infected with RFP-SL1344. Intracellular replication was monitored by measuring the relative area occupied by RFP-fluorescence in **(B)** infected cells and colony forming unit **(C)** assay. (*N* = 3, Error bar denote mean ± S.D., Scale bar 20 μm, ^*^p < 0.05, ^**^p < 0.005).

Because MTMR4 was recruited to the SCV and modulation of its level of expression impacted on SCV early maturation, we next determined if specific depletion of MTMR4 had an impact on intracellular *Salmonella* growth. Cells depleted for MTMR4 were infected with RFP-SL1344 and prepared for immunofluorescent investigation or harvested for colony forming unit (CFU) assays at specific times post-infection (Figure 4). No significant difference was observed 1 h p.i, indicating that depletion of MTMR4 does not impair *Salmonella* invasion. A significant reduction in viable bacteria was observed in the MTMR4 knockdown cells as early as 4 h p.i through to 12 h p.i. (Figure 4C). Immunofluorescent analysis confirmed a decrease in total number of intracellular *Salmonella* in MTMR4 depleted cells 12 h p.i (Figures 4A,B). The apparent incongruence between the CFU and immunofluorescence assays earlier in the infection suggests that a proportion of the *Salmonella* in MTMR4-depleted cells are no longer viable.

### Depletion of MTMR4 increases recognition of SCV by autophagy

**Figure 5:**
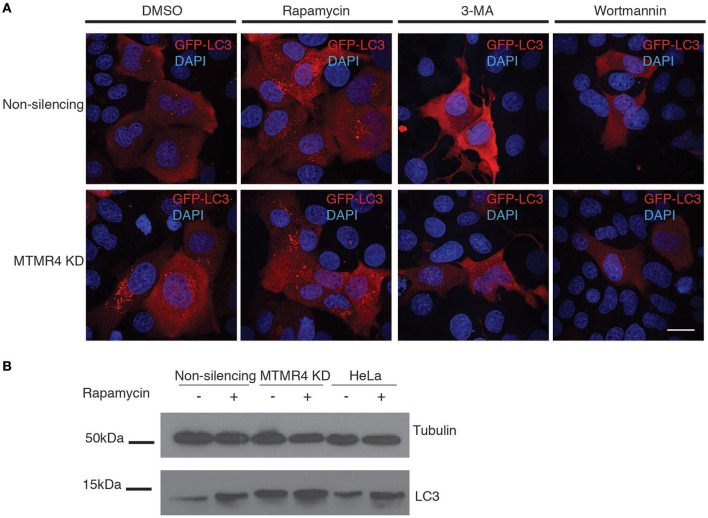
**Depletion of MTMR4 enhances autophagy. (A)** MTMR4-depleted HeLa and control cells were transfected with GFP-LC3, and treated with DMSO, 100 nM rapamycin, 100 nM wortmannin, or 20 mM 3-MA. Coverslips were then fixed and immunolabelled using antibodies against GFP. **(B)** Representative blot of MTMR4 knockdown and control whole cell lysates were harvested and 20 μg of proteins subjected to western immunoblotting against LC3 and tubulin. Scale bar 20 μm.

Ordinarily, *Salmonella* maintains the SCV such that it matures, acquires LAMP1 from 1 h p.i and and forms SIFs that expand the compartment to accommodate the replicating bacteria within. Birmingham et al. (2006) reported that a small subpopulation of *Salmonella* is unable to establish such a stable replicative niche, and are exposed to the cytoplasm after damaging the SCV with their SPI1 components. The exposed bacteria are consequently recognized and destroyed by the cell's autophagic system (Birmingham et al., 2006). Several active MTMR family members have recently been implicated as negative regulators of autophagy (Walker et al., 2001; Vergne et al., 2009; Zou et al., 2012). As such, we questioned if the bactericidal effect observed in MTMR4-depleted cells could be attributed to elevated autophagy. This was confirmed by both western immunoblotting of endogenous LC3 and fluorescence microscopy of GFP-LC3. Because the shRNA constructs used to generate non-silencing and MTMR4 knockdown cells also encode turbo-GFP, antibodies against the GFP-fused to LC3 and appropriate secondary antibodies were used to monitor the subcellular distribution of GFP-LC3 rather than its native fluorescence. In both cases, MTMR4 knockdown cells presented elevated autophagy when compared to the control cells (Figure 5A) as well as the increased endogenous LC3 band intensity by western immunoblotting (Figure 5B). Within HeLa cells, the accepted non-phagocytic model used in *Salmonella* research, LC3 is observed as a single band with the soluble form of LC3-II not observed (Klionsky et al., 2016). Therefore, we are not able to directly monitor variations in the ratio of soluble LC3 with lipidated LC3-II to monitor autophagic flux in these cells. To verify the identity of the GFP-LC3-positive puncta, cells were treated with inhibitors of autophagy (wortmannin or 3-methyladenine (3-MA); Lindmo and Stenmark, 2006) and inducers of autophagy (rapamycin; Jung et al., 2010). Consistent with expectation, the number of GFP-LC3 positive puncta observed in the MTMR4 knockdown cells was decreased when autophagy was inhibited and the number of GFP-LC3 puncta within control cells rose to meet that present in DMSO-treated MTMR4 knockdown cells when autophagy was induced with rapamycin. The loss of GFP-LC3 punctate structures following treatment with wortmannin is consistent with a global increase in autophagy under resting conditions in MTMR4 knockdown cells. We speculate that this elevates the defensive potential of these cells making the environment less hospitable to the pathogen.

**Figure 6:**
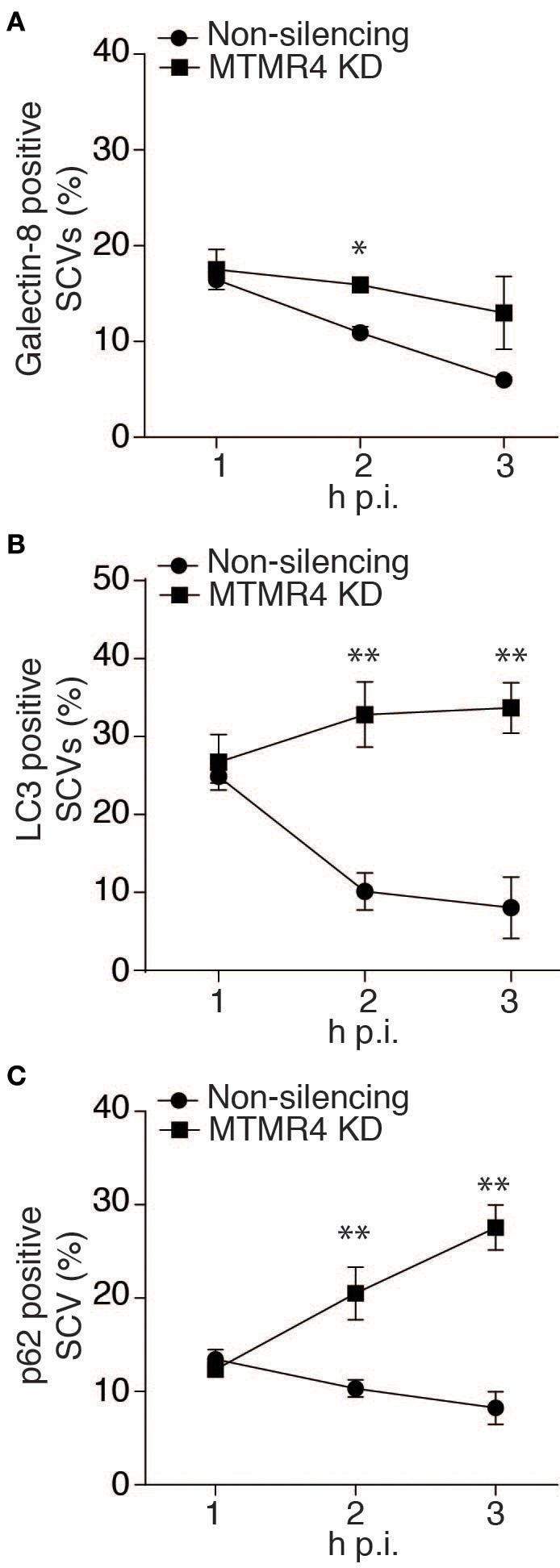
**The proportion of damaged SCVs increases in MTMR4 depleted cells**. MTMR4-depleted A431 cells and non-silencing control cells were infected with RFP-SL1344 or WT-SL1344. Cells were fixed at stipulated time points and probed for association autophagic markers. Quantification of the percentage of **(A)** galectin-8+, **(B)** recruitment of GFP-LC3+ or **(C)** p62+ bacteria. (*N* = 3, Error bar denote mean ± S.D., ^*^p < 0.05, ^**^p < 0.005).

We subsequently extended these observations into the context of *Salmonella* infection. First, we determined the association rate of galectin-8, a β-galectoside-binding lectin that has been shown to accumulate on damaged bacteria-containing vacuoles (Thurston et al., 2012). A time course assay on *S. typhimurium*-infected MTMR4-depleted cells was performed and probed for endogenous levels of galectin-8 associated with the SCV using specific antibodies. Although, there were no differences between MTMR4 depleted and control cells at 1 h p.i, at 3 h p.i, galectin-8 positive SCVs in knockdown cells were observed at higher frequency when compared to the proportion of galectin-8 positive SCVs in control cells which rapidly dropped to background levels (~5%; Figure 6A).

Having shown an increased proportion of damaged SCVs in MTMR4 depleted cells, we next wanted to quantify the number of SCVs being recognized by the autophagy pathway. To achieve this, MTMR4-depleted cells infected with RFP-SL1344 or WT-SL1344 were examined to determine the proportion of SCVs positive for GFP-LC3 or endogenous p62. Similar to what was observed with galectin-8, when compared with control cells, there was an increase proportion of SCVs with both LC3 and p62 in MTMR4 depleted cells (Figures 6B,C). LC3 association with the SCV remained elevated (~30%) in MTMR4-depleted cells relative to control cells from 2 to 3 h p.i. The association of p62 with SCVs rapidly increased from 1 h p.i. (~13%) to 3 h p.i (~27%). Taken together, these findings demonstrate that the depletion of MTMR4 leads to an increased number of SCVs being damaged and the bacteria being targeted by the host cell's autophagic pathway.

## Discussion

The SCV undergoes a maturation process similar to that of an endosome, but stops short of fusing with lysosomes (Eswarappa et al., 2010). To achieve this, *Salmonella* modulates host cell phosphoinositide metabolism to alter the cell's membrane trafficking pathways(Kerr et al., 2012). Previously, we demonstrated that the SCV is transiently enriched for PI(3)P and that perturbation of phosphatidylinositide 5-kinase activity interferes with the later stages of SCV maturation and the intracellular replication of the pathogen (Kerr et al., 2010). Here we go onto demonstrate the accumulation of PI(3,5)P_2_ on the maturing SCV and set out to investigate the potential role for PI(3)-phosphatases in SCV maturation.

MTMR4 is one of a large family of 3-phosphatases that have been reported to hydrolyse PI(3)P and PI(3,5)P_2_ (Lorenzo et al., 2006; Naughtin et al., 2010). We find that when MTMR4 is ectopically expressed, the proportion of PI(3)P-positive endosomes, as monitored by recruitement of EEA1, decreases while RNAi mediated depletion of MTMR4 leads to a corresponding increase in the proportion of EEA1-positive endosomes. No change in PI(3,5)P_2_-positive endosomes, as monitored using a PI(3,5)P_2_-specific probe (Li et al., 2013) was observed. This is potentially due to PI(3)P being the preferential substrate of MTMR4 or alternatively MTMR4 membrane recruitement via its C-terminal FYVE domain (Lorenzo et al., 2006) precludes it from been associated with PI(3,5)P_2_ positive membranes.

Interestingly, despite MTMR3 and MTMR4 each encoding a FYVE domain (Lorenzo et al., 2005, 2006), only MTMR4 was observed to be recruited to the nascent SCV. This recruitment was dependent upon PI(3)P as ΔSopB *Salmonella* mutants which are unable to generate PI(3)P on their SCVs through the recruitment of the class III PI 3-kinase, Vps34 (Hernandez et al., 2004; Mallo et al., 2008), did not recruit MTMR4. Additionally, no association between MTMR4 and the SCV was observed in the later stages of the infection when PI(3)P levels are much reduced (Kerr et al., 2010).

Strikingly, we find that depletion of MTMR4 destabilized the SCV and led to a concomitant depletion in intracellular bacterial viability. Multiple studies have offered explanations as to why *Salmonella* chooses to reside within a vacuole and modulate trafficking instead of proliferating freely in the nutrient rich cytosol. Perhaps most compelling amongst these is that the SCV provides the means to avoid the myriad of host defenses that serve as a protective net for the host cell in the event of an infection (Nakagawa et al., 2004; Birmingham et al., 2006; Thurston et al., 2012). Autophagy in particular has been demonstrated to target cytosolic *Salmonella* for degradation within autophagolysosomes (Birmingham et al., 2006). A recent siRNA-based screen determined that depletion of MTMR6, MTMR7, and MTMR14 lead to increased levels of autophagic vacuoles as well as an upregulation of long-lived protein proteolysis (Vergne et al., 2009). MTMR3 has also been implicated in regulating constitutive autophagy initiation and autophagosome size in epithelial cells (Taguchi-Atarashi et al., 2010). It is therefore perhaps not so surprising that depletion of MTMR4, which induces increased cellular concentrations of PI(3)P-positive organelles, also leads to enhanced autophagy. This enhanced state of autophagy likely contributes to the increased capacity of the MTMR4-depleted cells to eliminate intracellular *Salmonella*. Recent studies have made conflicting conclusions about the role of autophagy with regards to the intracellular viability of *Salmonella* (Yu et al., 2014; Kreibich et al., 2015). Using CFU assays to monitored viable bacteria 6 h post-infection, Yu et al. (2014) observed decreased viability in HeLa cells when autophagy was inhibited using RNAi-depletion of LC3 while Kreibich et al. (2015) observed elevated viable bacteria in autophagy impaired Atg5−/− MEFs. The differences between these studies may reflect multiple functions associated with the individual autophagy components manipulated, cell types used or the differences in MOI used.It should also be noted that these targeted factors demonstrated to directly regulate autophagy whilst, as demonstrated in our findings, MTMR4 regulates both cellular PI(3)P and autophagy, both of which are recognized to be intrinsic to intracellular survival of *Salmonella*.

Taken together we propose a model where by PI(3)P turnover on the SCV is a tightly regulated process coordinated through the action of both bacterial effectors like SopB as well as the host factors, PIKfyve and MTMR4. Disregulation of this turnover through MTMR4-depletion both destabilizes the SCV and elevates cellular autophagy leading to destruction of the pathogen. MTMR4 is therefore a novel host factor that plays a vital role in *Salmonella* pathogenesis.

## Author contributions

All authors contributed to the conception, design, interpretation of the findings, drafting, and preparation of the manuscript. WT and MK executed the experiments.

## Funding

This work was supported by funding from the National Health and Medical Research Council (NHMRC) of Australia (606788). MK is supported by an Australian Research Council Discovering Early Career Researcher Award (DE120102321). RT is supported by NHMRC Senior Research Fellowship (APP1041929).

### Conflict of interest statement

The authors declare that the research was conducted in the absence of any commercial or financial relationships that could be construed as a potential conflict of interest.
